# Meta-analysis for genome-wide association studies using case-control design: application and practice

**DOI:** 10.4178/epih.e2016058

**Published:** 2016-12-18

**Authors:** Sungryul Shim, Jiyoung Kim, Wonguen Jung, In-Soo Shin, Jong-Myon Bae

**Affiliations:** 1 Institute for Clinical Molecular Biology Research, Soonchunhyang University Hospital, Seoul, Korea; 2 Department of Radiation Oncology, Ewha Womans University School of Medicine, Seoul, Korea; 3 Department of Education, Jeonju University, Jeonju, Korea; 4 Department of Preventive Medicine, Jeju National University School of Medicine, Jeju, Korea

**Keywords:** Meta-analysis, Reviews, Genome-wide association study, Polymorphism, Genetic models

## Abstract

This review aimed to arrange the process of a systematic review of genome-wide association studies in order to practice and apply a genome-wide meta-analysis (GWMA). The process has a series of five steps: searching and selection, extraction of related information, evaluation of validity, meta-analysis by type of genetic model, and evaluation of heterogeneity. In contrast to intervention meta-analyses, GWMA has to evaluate the Hardy–Weinberg equilibrium (HWE) in the third step and conduct meta-analyses by five potential genetic models, including dominant, recessive, homozygote contrast, heterozygote contrast, and allelic contrast in the fourth step. The ‘genhwcci’ and ‘metan’ commands of STATA software evaluate the HWE and calculate a summary effect size, respectively. A meta-regression using the ‘metareg’ command of STATA should be conducted to evaluate related factors of heterogeneities.

## INTRODUCTION

Malignant neoplasm, or cancer, is one of the most prevalent chronic diseases, which develops as a result of a somatic mutation. Advancing from this theory, a personalized medicine is currently gaining traction for the diagnosis and treatment of cancer [1], and such trends call for the synthesis of evidence related to genome-wide epidemiology [2].

With the advances in genetic technologies, the subjects of analyses in studies aiming to discover disease-related genomes have changed into chromosomal abnormalities, allelic heterogeneity, and single nucleotide polymorphisms (SNPs). According to these changes, linkage analysis studies, genetic association studies (GTAS), and genome-wide association studies (GWAS) has been currently ongoing [2,3].

However, a phenomenon known as the “winner’s curse,” which is characterized by low replicability of results, has been appearing in follow-up studies on genes that were previously associated with a particular disease through genome-wide epidemiology studies [4-6]. Population stratification, diverse testing methods, and insufficient sample sizes have been implicated in this phenomenon [7-9], all of which constitute the rationale for the meta-analysis of genome-wide epidemiology studies [10-12].

This review introduces the process of a genome-wide meta-analysis (GWMA), which involves a meta-analysis of findings of GWAS that investigate the SNPs associated with a particular disease [13]. Particularly, this study presents an example of a meta-analysis in practice, in an attempt to inspire further GWMA studies in Korea.

## PROCESS OF GENOME-WIDE META-ANALYSIS

The general procedures of a GWMA introduced by previous studies [10,12-17] could be divided into five steps as shown in Table 1. Two features that distinguish GWMA from traditional systematic reviews are the Hardy-Weinberg equilibrium (HWE) test in step 3 for a quality evaluation of the selected literature and the use of genetic models for meta-analyses in step 4.

Here, we present the study by Song et al. [18], which examined the association between Fc receptor-like 3-169 C/T polymorphism and rheumatoid arthritis in Asians, to describe the process of HWE testing and summary effect size calculating using a statistical program. The study selected 15 articles with a pooled sample of 22,312 individuals (11,170 cases + 11,142 controls). The selected articles were divided into three races (Asians, Europeans, and Native North Americans) for subgroup analysis. The polymorphic genotypes for the meta-analysis were CC, CT, and TT. We introduce the commands used on STATA version 14.2 (StataCorp, TX, USA) and interpret the results.

### Step 1: searching and selection

The search for GWAS articles involves different sources and keywords from those used for a search of general systematic reviews. We recommend the use of data sources on the organized tables by Casado-Vela et al. [19], Ramasamy et al. [20], and Wallace et al. [21]. Keywords such as ‘genetics, alleles, and polymorphisms’ are some medical subject headings regarding genome-wide epidemiology [22].

We recommend the use of the flow chart suggested by Sagoo et al. [12] for the literature selection process following the electronic search.

### Step 2: extraction of related information

The sets of information extracted from the selected GWAS articles are needed for the evaluation of the validity of each article in the next step. Items for evaluating the validity of GWAS articles have been suggested by Attia et al. [4], de Bakker et al. [14], Ramasamy et al. [20], and Khoury et al. [23]. Considering that GWMA results are applied to patient treatments, we strongly recommend the use of the items suggested by Attia et al. [4]. The organization of tables is recommended by the suggestions of Sagoo et al. [12].

If the quality of each of the selected genetic epidemiology studies must be assessed, the assessment checklist provided as supplementary data in the study by Thakkinstian et al. [24] or the checklist suggested on the “Strengthening the Reporting of Genetic Association Studies” by Little et al. [25] may be used.

### Step 3: evaluation of validity

One critical aspect of validity assessment for GWMA findings is the satisfaction of HWE assumption. HWE states that the frequencies of genes and genotypes remain in equilibrium over generations under limited conditions [3]. For example, given that the frequencies of two alleles, called A and a, of a gene are p and q, respectively, where p+q=1, the frequencies of the genotypes AA, Aa, and aa are p^2^, 2pq, and q^2^, respectively, where p²+2pq+q²=1. Using this equation, we can predict the frequency of a genotype with a known allele frequency.

The subjects of HWE testing depend on the study design. In a cohort study or cross-sectional study, HWE should be tested on the entire study population. On the other hand, HWE is only tested on the control group in a case-control study because the case group may not confirm to the HWE if the genotype is associated with a disease. Studies that deviate from the HWE should be excluded from step 4, and their meanings should be investigated in step 5 through a sensitivity analysis.

The most popular test to verify the HWE is the chi-squared test [26], a statistical technique that compares the observed values from a group with estimated values based on the assumption of HWE. In other words, it assesses the degree of deviation of observed values from the estimated values. A p-value of less than 0.05 is considered statistically significant and is interpreted to be a violation of the HWE.

**Figure 1.**
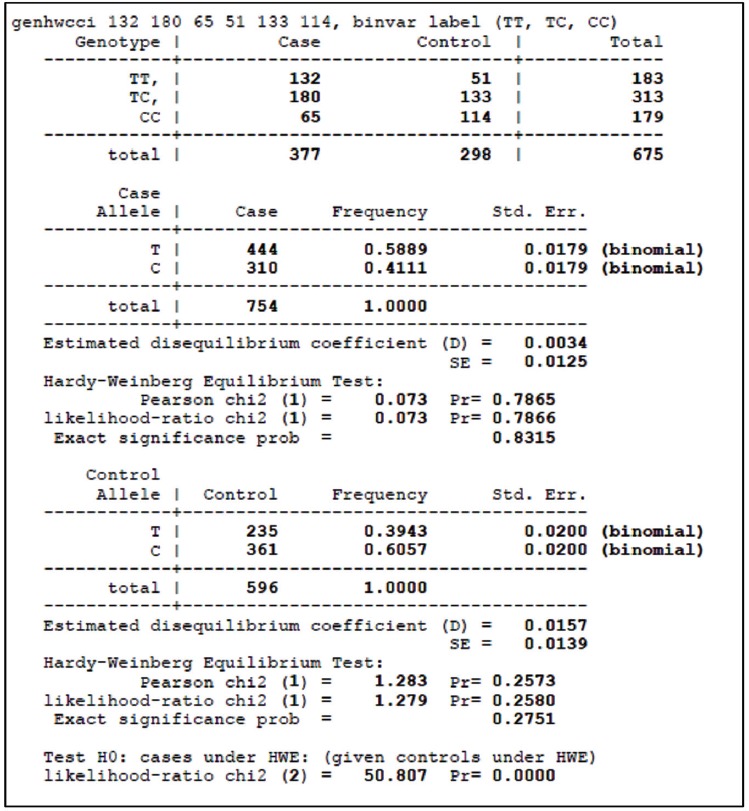
Results of Hardy-Weinberg equilibrium testing using the STATA ‘genhwcci’ command of Han et al. [27].

For HWE analysis of case-control studies in the STATA software, genotypic counts of the case and control groups should be listed following the <genhwcci> command. For example, in Table 1 of the article by Song et al. [18], the genotypic counts for TT, TC, and CC in one of the 15 studies (Han et al. [27]) were 132, 180, and 65 in the case groups and 51, 133, and 114 in the control groups, respectively. Figure 1 shows the results of entering <genhwcci 132 180 65 51 133 114, binvar label (TT, TC, CC)> into the software. ‘binvar’ requests that standard errors from a binomial distribution are reported, and ‘label’ requests that results are presented according to the genotype. The p-value in the chi-square test for the control group was 0.257, which indicates that it does not violate the HWE.

### Step 4: meta-analyses by types of genetic model

In a C/T polymorphism where C is dominant and T is recessive, there are five possible types of genetic models: dominant (CC+CT vs. TT), recessive (CC vs. CT+TT), homozygote contrast (CC vs. TT), heterozygote contrast (CC vs. CT), and allelic contrast (C vs. T) [17,18,28.29].

Add the frequencies for the case and control groups of each article according to each model before performing the meta-analyses. For example, in the study by Han et al. [27], multiply CC and TT by two and add TC to each value for an allelic contrast (C vs. T) (Figure 1). In other words, the C for the case group becomes 310 (=65 [CC]×2+180 [TC]), and T becomes 444 (=132×2+180). By the same method, the C for the control group becomes 361 (=114×2+133), and T becomes 235 (=51× 2+133). Apply this method to the remaining 14 articles, and perform the meta-analyses.

**Figure 2.**
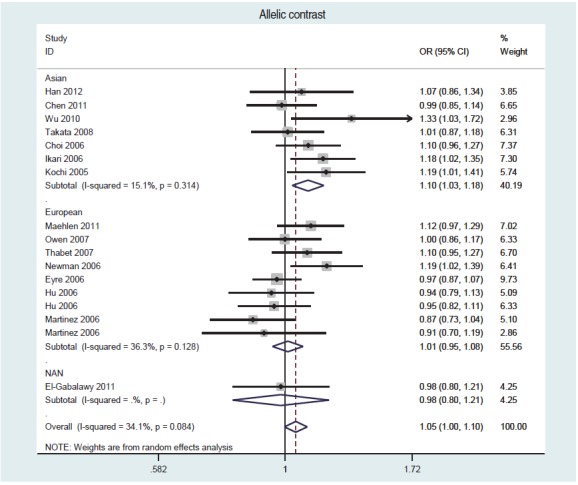
A forest plot of an alleleic contrast model, using the STATA ‘metan’ command of Song et al. [18]. OR, odds ratio; NAN, North American Natives; CI, confidence interval.

For a frequency-based meta-analysis on STATA, use the <metan> command. Refer to Shim et al. [30] for creating a forest plot, calculating summary effect size, calculating the I-squared value for an evaluation of heterogeneity, creating a funnel plot to assess publication bias, and applying options for the Egger or Begg test. Figure 2 is a forest plot obtained from a meta-analysis of an allelic contrast model with the data from Song et al. [18], using the command <metan case_C case_T control_C control_T, or randomi by(ethnicity)>.

### Step 5: evaluation of heterogeneity

If heterogeneity is present, difference of race should be first considered [15,29], as differences in genetic pools may lead to heterogeneity among genome-wide epidemiology studies [4,31]. Hence, Song et al. [18] performed subgroup analyses by dividing the subjects into three races: Asians, Europeans, and Native North Americans. In addition, differences in allele frequencies may also induce heterogeneity among studies [32].

If heterogeneity is determined to persist, a random effect model may be applied [33,34]. However, a meta-regression may be applied to identify the cause of the heterogeneity [29,35]. Meta-regression is recommended only for analysis of ten or more articles, and its STATA command is <metareg> [30].

## CONCLUSION AND SUGGESTIONS

Two features that distinguish GWMA from the intervention meta-analyses are that GWMA uses HWE to verify the validity of a study and performs meta-analyses according to the five possible types of genetic models.

If individual patient data, as opposed to the findings of the selected literature, are used, the STATA <metagen> command may be used [36]. Furthermore, there may be a hypothesis in which the outcome variables are continuous and not dichotomous. A case in point is the investigation of differences in bone density according to vitamin D receptor polymorphisms [17]. We plan to describe the process of GWMA involving continuous outcome variables in a future article. In addition, we shall introduce genome search meta-analysis (GSMA), which was developed for meta-analysis for ordinal outcome variables [37], at another time.

Currently, genome-wide epidemiology is evolving into system epidemiology using multi-omics, including proteomics, metabolomics, and epigenomics, in pursuit of precision medicine [19,38,39]. Amid this trend, GWMA is vital in that it can reinterpret existing studies and suggest future research directions. We hope this article provides inspiration for further studies.

## Figures and Tables

**Table 1.** Five steps of conducting a genome-wide meta-analysis

	Actions
Step 1	Searching and Selection
Step 2	Extraction of related information
Step 3	Evaluation of validity
Step 4	Meta-analyses by types of genetic model
Step 5	Evaluation of heterogeneity
