# Supplementary material for: Meta-analysis for genome-wide association studies using case-control design: application and practice
Source: Epidemiol Health. 2016 Dec 18;38:e2016058. doi: 10.4178/epih.e2016058 (PMC5309730; doi:10.4178/epih.e2016058)
Supplement: Supplementary file 1 [file epih-38-e2016058-supplementary.pdf]

<방법론 단신>

유전체 연관 환자-대조군 연구의 메타분석: 적용과 실제

Meta-analysis for Genome-wide Association Studies using Case-Control Design: Application and Practice

Abstract

This review aimed to arrange the process of a systematic review for genome-wide association studies in order to practice and apply the genome-wide meta-analysis (GWMA). The process has a series of 5 steps: searching and selection, extraction of related information, evaluation of validity, meta-analysis by types of genetic model, and evaluation of heterogeneity. Contrast to intervention meta-analysis, GWMA has to evaluate Hardy–Weinberg equilibrium (HWE) on third step and to conduct meta-analyses by five potential genetic models such as dominant, recessive, homozygote contrast, heterozygote contrast and allelic contrast on fourth step. The 'genhwcci' and 'metan' commands of STATA software evaluates HWE test and estimates a summary effect size, respectively. A meta-regression using 'metareg' command of STATA would be conducted to evaluate related factors of heterogeneities.

Keywords: Meta-analysis, Reviews, Genome-wide association study, Polymorphism, Genetic models

서론

대표적인 만성질환인 악성종양 (malignant neoplasm) 즉 암 (cancer)는 체세포 돌연변이 (somatic mutation) 으로 발생된다. 이 이론에서 발전하여 오늘날은 암의 진단 및 치료에 있어 환자 개인맞춤의학 시대로 진입하고 있다 [1]. 이런 흐름에 유전체 역학연구 (genome-wide epidemiology)와 관련한 근거의 생성을 크게 요구하고 있다 [2].

질병발생과 관련한 유전체를 찾는 연구의 분석 대상은 유전기술의 발달에 따라 염색체 이상 (chromosomal abnormalities), 대립 유전자의 이형성 (allelic heterogeneity), 단일염기다형성 (single nucleotide polymorphism, SNP)으로 변천해 왔으며, 각 분석대상에 맞추어 linkage analysis study, genetic association study (GTAS), genome-wide association study (GWAS) 이름의 유전체역학연구가 활발히 수행되고 있다 [2,3].

그러나 유전체역학연구를 통해 질병과 관련하였다고 보고한 유전자들 중 후속연구에서 재현성 (replication) 이 떨어지는 '승자의 저주 (winner's curse)' 란 현상을 보이고 있다 [4-6]. 그 이유에 대하여는 인종간의 유전체 다양성 (population stratification), 다양한 검사 측정법, 부적절한 분석 대상수 등으로 이 현상을 해석하고 있지만 [7-9], 이러한 이유들은 곧 유전체역학연구에서 메타분석의 필요성을 부각시키는 근거가 된다 [10-12].

본 원고는 특정 질병과 연관된 SNP를 알아보는 GWAS의 연구 결과들에 대하여 메타분석을 적용한 '유전체 메타분석 (Genome-wide Meta-analysis, GWMA) [13]의 연구 전체 과정을 소개하고, 특히 메타분석의 실제 적용의 예를 제시하여서 국내 GWMA 연구의 활성화를 도모하는 것을 목표로 한다.

본론

GWMA의 일반적인 수행 과정을 소개한 기존의 논문들 [10, 12-17]의 내용을 재정리하면, 표 1처럼 5 단계로 정리할 수 있다. 기존의 체계적 고찰과 달리 유전체 메타분석이 차별화 되는 것은 셋째 단계로 선정된 논문의 질적 평가를 위해 Hardy-Weinberg 평형상태 (Hardy-Weinberg Equilibrium, HWE)를 확인하는 점과 넷째 단계로 유전 모델 (genetic model)을 반영하여 메타분석을 수행한다는 점이다.

통계프로그램을 적용하여 HWE 검정과 요약효과크기 (summary effect size)를 얻어내는 것을 설명하기 위하여 아시아인에 있어서 Fc receptor-like 3 (FCRL3)-169 C/T polymorphism와 rheumatoid arthritis의 관련성을 연구한 Song et al. [18]을 예제로 삼았다. 해당 메타분석의 최종 선정된 분석 대상은 총 15개 논문이었으며, 전체 대상자는 22,312명 (= 환자군 11,170 + 대조군 11,142)이었다. 하부군 분석을 위하여 아시아인, 유럽인, 북미원주민의 3 인종으로 구분하였다. 분석 대상인 다형성 유전형 (polymorphic genotypes)은 CC, CT, TT 이었다. 이후 통계분석 적용을 위하여 STATA version 14.2 (StataCorp, TX, USA)의 명령어를 소개하고 그 결과를 해석하고자 한다.

1단계: 관련 논문 검색

일반적인 체계적 고찰 (systematic reviews)의 검색과정과 비교할 때, GWAS 논문의 검색과정은 검색 자료원과 중심단어 (keywords)가 다를 뿐이다. GWAS 검색자료원은 Casado-Vela et al. [19], Ramasamy et al. [20], Wallace et al. [21] 의 표로 정리한 자료원 (data sources) 을 활용하기를 권한다. 유전체 역학과 관련하여 'genetics, alleles, polymorphisms' 의 중심단어가 medical

subject headings 으로 선정되어 있다 [22].

전자 검색을 한 이후, 관련 논문의 선정과정을 밝히는 흐름도는 Sagoo et al. [12] 가 제시한 것을 사용할 것을 권한다.

2단계: 선정한 논문의 관련 정보 추출

선정된 GWAS 논문들에서 추출할 정보들은 다음 단계인 각 논문의 타당성 평가를 위해 필요한 것들이다. GWAS 논문의 타당성을 평가하는 항목으로는 Attia et al. [4], de Bakker et al. [14], Ramasamy et al. [20], Khoury et al. [23] 논문들에서 표로 정리하여 제시하고 있다. 이중 GWMA 결과를 환자 치료에 활용한다는 점을 반영한다면, Attia et al. [4] 이 제시한 항목들을 활용하기를 적극 권한다. 이 과정에서 추출한 정보의 표 정리는 Sagoo et al. [12] 가 제시한 것을 사용할 것을 권한다.

만약 추출과정을 통해 선정된 유전역학 관련 논문들 각각에 대한 질적 수준을 평가할 필요성이 있다면, Thakkinstian et al. [24]이 부록으로 제시한 평가표나 유전체 역학연구결과에 대한 보고지침 (strengthening the reporting of genetic association studies)의 점검표를 적용할 수 있다 [25].

3단계: 추출 정보들에 대한 타당성 평가

GWMA 연구결과에 대한 타당성 평가의 핵심은 HWE 여부이다. HWE란 제한된 조건하에서 유전자와 유전자형의 빈도가 세대를 거듭하여도 변하지 않고 항상 평형을 이룬다는 법칙이다

[3]. 예를 들어, 유전자 A의 빈도가 p, 대립유전자 a의 빈도가 q의 값을 가지며 p+q=1의 값을 갖는다고 할 때, population에서 평형을 이룬다면 세 가지 유전자형 AA, Aa, aa의 유전자형의 빈도는 각각 p^2, 2pq, q^2이 되며, $p^2+2pq+q^2=1$이 된다. 이를 이용하여 대립유전자의 빈도를 알면 유전자형의 빈도를 예측할 수 있다.

HWE 여부를 확인하는 대상은 연구설계 (study design)에 따라 달라진다. 코호트 연구 (cohort study) 또는 단면조사연구(cross-sectional study)일 때는, 연구의 대상이 되는 전체 그룹을 대상으로 HWE를 확인한다. 반면에 환자-대조군 연구 (case-control study) 경우는 대조군에 대해서만 HWE를 평가한다. 그 이유는 환자군의 경우 유전자형(genotype)이 질병과 연관되어 있다면 HWE란 평형상태를 이루지 않을 수 있기 때문이다. HWE를 만족하지 않는 연구들은 4 단계의 메타분석에서 제외하고 수행하는 것이 타당하며, 5 단계에서 민감도 분석을 수행하여 그 의미를 찾기를 제안한다.

HWE 여부를 확인하는 주된 검정법은 카이자승법 (chi-squared test)이다 [26]. 이는 확인대상 집단에서 얻어낸 관측 결과와 HWE 가정에 따라 얻을 수 있는 예측 결과를 비교하는 통계기법이다. 다시 말해서 관측치와 예측치간의 편차 (deviation)의 정도를 평가하며, 카이자승 분포상 p값이 0.05보다 작으면 통계학상 유의한 차이가 있다고 보며, HWE를 위배한다고 해석한다.

STATA 프로그램에서 환자-대조군 연구결과의 HWE 분석을 위해서는 <genhwcci> 명령어 다음에 환자군과 대조군의 유전형 숫자를 나열하면 된다. 예를 들어 Song et al. [18]에서 제시한 표 1에서, 선정한 15개 논문 중의 하나인 Han et al. [27]의 TT, TC, CC 유전형 (genotype)을 보인 경우는 환자군 132, 180, 65명과 대조군 51, 133, 114명 이었다. 따라서 <genhwcci 132 180 65 51

133 114, binvar label (TT, TC, CC)> 라고 입력한 결과는 그림 1과 같다. binvar 란 옵션은 binomial 분포를 이용한 표준오차를 추가로 구하라는 뜻이며, label 은 제시한 유전형에 맞추어 결과를 제시하라는 것이다. 대조군에서 Pearson 카이자승법의 p-value가 0.257로 HWE를 위배하지 않는다고 해석하게 된다.

4단계: 적합한 유전모델 결정

C/T polymorphism에서 C를 우성으로, T를 열성으로 간주할 때, 조합 가능한 유전 모델은 우성 (dominant; CC+CT vs TT), 열성 (recessive; CC vs CT+TT), 동형접합자 대조 (homozygote contrast, CC vs TT), 이형접합자 대조 (heterozygote contrast, CC vs CT), 대립유전자 대조 (allelic contrast, C vs T)의 5가지로 나눌 수 있다 [17,18,28.29].

각 모델에 맞추어 개별 논문의 환자군과 대조군의 빈도수를 합산한 다음 메타분석을 준비한다. 예를 들어 대립유전자 대조 (C vs T)를 위하여 Han et al. [27]의 CC, TT 군에 두 배를 한 다음 TC 값을 각각 더한다 [Fig 1]. 즉 환자군의 C는 CC군 65의 두배인 130에 TC 180을 더한 310 (=65×2+180) 이 되며, T는 444 (=132×2+180) 가 된다. 같은 방법으로 대조군의 C는 361 (=114×2+133), T는 235 (=51×2+133) 이다. 이런 작업을 나머지 14개 논문에 적용한 다음, 메타분석을 수행한다.

빈도수를 활용한 메타분석을 위해 STATA에서 <metan> 명령어를 사용한다. 숲그림 (forest plot) 작성, 요약한 효과 크기 (summary effect size) 산출, 이질성 수준을 평가하기 위한 I-squared 값 산출, 출판바이어스 (publication bias) 수준을 평가하는 깔대 그래프 (funnel plot) 작

성과 Egger 혹은 Begg 검정을 위한 metan 명령문의 옵션 사용은 Shim et al. [30]를 참조하기를 권한다. 그림 2는 Song et al. [18] 의 추출 정보를 이용하여 대립유전자 대조 유전모델에 대한 메타분석을 시행하기 위하여 <metan case_C case_T control_C control_T, or randomi by(ethnicity)> 라는 명령어를 주어 얻은 Forest plot 이다.

5단계: 이질성 평가

이질성이 확인이 되면 우선적으로 인종의 차이를 고려해야 할 것이다 [15,29]. 유전자 풀 (genetic pool)이 다르면 유전체 역학연구들 간에는 이질성이 생길 수 있기 때문이다 [4,31]. 따라서 Song et al. [18]에서도 아시아인, 유럽인, 북미원주민의 3 인종으로 구분하여 하부군 분석 (subgroup analysis)를 시행하였다. 또한 대립유전자의 빈도가 논문들 간에 차이가 있으면 이질성을 보일 수도 있다 [32]

만약 이질성이 여전히 있다고 판단이 되면 무작위효과 모형 (random effect model)을 적용해 볼 수 있지만 [33,34], 이질성 원인을 파악하기 위하여 메타회귀법 (meta-regression)을 적용할 수 있다 [29,35]. 메타회귀는 분석대상 논문이 10개 이상일 때 추천되며, STATA 명령어는 <metareg> 이다 [30].

결론 및 제언

유전체 메타분석의 수행과정에서 기존의 중재 (intervention) 메타분석과 다른 점은 논문의 타당성을 HWE 검정으로 확인한다는 점과, 가능한 5가지 유전모델에 맞추어 메타분석을 수행

한다는 점이다.

이번처럼 메타분석의 대상이 선정된 논문의 결과가 아니라, 개인별 자료 (individual patient data)를 활용하는 것이라면 STATA의 <metagen> 명령어를 활용할 수 있다 [36]. 또한, 가설에 따라서는 결과변수가 이분형이 아니라, 연속형일 경우가 있을 수 있다. 비타민 D 수용체의 다형성에 의한 골밀도 차이를 알아본 것이 하나의 예가 된다 [17]. 이렇게 연속형 결과변수를 갖는 유전체 메타분석에 대하여는 다른 논문으로 설명을 할 예정이다. 또한 결과변수가 순위형일 경우의 메타분석을 위하여 개발된 Genome Search Meta-analysis (GSMA) [37]도 다른 기회를 빌어 소개코자 한다.

현재 유전체 역학은 정밀의학 (precision medicine)을 추구하기 위하여 단백체학 (proteome), 대사체학 (metabolome), 후생유전학 (epigenome) 등의 다학제 (multi-omics)를 활용한 시스템 역학 (system epidemiology)로 진화하고 있다 [19,38,39]. 이런 흐름 속에 유전체 메타분석 연구는 기존의 연구들을 재해석하고, 향후 연구 방향을 제시한다는 점에서, 향후 활발히 수행할 필요가 있겠다. 본 원고가 이에 일조하기를 기대한다.

감사의 글

본 원고는 메타분석연구회(신인수 회장) 연구활동의 결과물임을 밝힙니다.

References

1. Jameson JL, Longo DL. Precision medicine--personalized, problematic, and promising. N Engl J Med 2015;372(23):2229-34.
2. McCarthy JJ, McLeod HL, Ginsburg GS. Genomic medicine: a decade of successes, challenges, and opportunities. Sci Transl Med 2013;5(189):189sr4.
3. Attia J, Ioannidis JP, Thakkinstian A, McEvoy M, Scott RJ, Minelli C, et al. How to use an article about genetic association: A: Background concepts. JAMA 2009;301(1):74-81.
4. Attia J, Ioannidis JP, Thakkinstian A, McEvoy M, Scott RJ, Minelli C, et al. How to use an article about genetic association: B: Are the results of the study valid? JAMA 2009;301(2):191-7.
5. Attia J, Ioannidis JP, Thakkinstian A, McEvoy M, Scott RJ, Minelli C, et al. How to use an article about genetic association: C: What are the results and will they help me in caring for my patients? JAMA 2009;301(3):304-8.
6. Lohmueller KE, Pearce CL, Pike M, Lander ES, Hirschhorn JN. Meta-analysis of genetic association studies supports a contribution of common variants to susceptibility to common disease. Nat Genet 2003;33(2):177-82.
7. Colhoun HM, McKeigue PM, Davey Smith G. Problems of reporting genetic associations with complex outcomes. Lancet 2003;361(9360):865-72.
8. Cardon LR, Bell JI. Association study designs for complex diseases. Nat Rev Genet 2001;2(2):91-9.
9. Ioannidis JP. Genetic associations: false or true? Trends Mol Med 2003;9(4):135-8.
10. Lee YH. Meta-analysis of genetic association studies. Ann Lab Med 2015;35(3):283-7.
11. Gwinn M, Ioannidis JP, Little J, Khoury MJ. Editorial: Updated guidance on human genome epidemiology (HuGE) reviews and meta-analyses of genetic associations. Am J Epidemiol 2014;180(6):559-61.
12. Sagoo GS, Little J, Higgins JP. Systematic reviews of genetic association studies. Human Genome Epidemiology Network. PLoS Med 2009;6(3):e28.
13. Zeggini E, Ioannidis JP. Meta-analysis in genome-wide association studies. Pharmacogenomics 2009;10(2):191-201.

14. de Bakker PI, Ferreira MA, Jia X, Neale BM, Raychaudhuri S, Voight BF. Practical aspects of imputation-driven meta-analysis of genome-wide association studies. Hum Mol Genet 2008;17(R2):R122-8.

15. Thompson JR, Attia J, Minelli C. The meta-analysis of genome-wide association studies. Brief Bioinform 2011;12(3):259-69.

16. Evangelou E, Ioannidis JP. Meta-analysis methods for genome-wide association studies and beyond. Nat Rev Genet 2013;14(6):379-89.

17. Thakkinstian A, McElduff P, D'Este C, Duffy D, Attia J. A method for meta-analysis of molecular association studies. Stat Med 2005;24(9):1291-306.

18. Song GG, Bae SC, Kim JH, Kim YH, Choi SJ, Ji JD, et al. Association between functional Fc receptor-like 3 (FCRL3) -169 C/T polymorphism and susceptibility to seropositive rheumatoid arthritis in Asians: a meta-analysis. Hum Immunol 2013;74(9):1206-13.

19. Casado-Vela J, Cebrián A, Gómez del Pulgar MT, Lacal JC. Approaches for the study of cancer: towards the integration of genomics, proteomics and metabolomics. Clin Transl Oncol 2011;13(9):617-28.

20. Ramasamy A, Mondry A, Holmes CC, Altman DG. Key issues in conducting a meta-analysis of gene expression microarray datasets. PLoS Med 2008;5(9):e184.

21. Wallace BC, Small K, Brodley CE, Lau J, Schmid CH, Bertram L, et al. Toward modernizing the systematic review pipeline in genetics: efficient updating via data mining. Genet Med 2012;14(7):663-9.

22. Attia J, Thakkinstian A, D'Este C. Meta-analyses of molecular association studies: methodologic lessons for genetic epidemiology. J Clin Epidemiol 2003;56(4):297-303.

23. Khoury MJ, Bertram L, Boffetta P, Butterworth AS, Chanock SJ, Dolan SM, et al. Genome-wide association studies, field synopses, and the development of the knowledge base on genetic variation and human diseases. Am J Epidemiol 2009;170(3):269-79.

24. Thakkinstian A, McEvoy M, Minelli C, Gibson P, Hancox B, Duffy D, et al. Systematic review and meta-analysis of the association between {beta}2-adrenoceptor polymorphisms and asthma: a HuGE review. Am J Epidemiol 2005;162(3):201-11.

25. Little J, Higgins JP, Ioannidis JP, Moher D, Gagnon F, von Elm E, et al. Strengthening the reporting of genetic association studies (STREGA): an extension of the STROBE statement. Eur

J Epidemiol 2009;24(1):37-55.

26. Wittke-Thompson JK, Pluzhnikov A, Cox NJ. Rational inferences about departures from Hardy-Weinberg equilibrium. Am J Hum Genet 2005;76(6):967-86.

27. Han SW, Sa KH, Kim SI, Lee SI, Park YW, Lee SS, et al. FCRL3 gene polymorphisms contribute to the radiographic severity rather than susceptibility of rheumatoid arthritis. Hum Immunol 2012;73(5):537-42.

28. Minelli C, Thompson JR, Abrams KR, Thakkinstian A, Attia J. The choice of a genetic model in the meta-analysis of molecular association studies. Int J Epidemiol 2005;34(6):1319-28.

29. Zintzaras E, Lau J. Synthesis of genetic association studies for pertinent gene-disease associations requires appropriate methodological and statistical approaches. J Clin Epidemiol 2008;61(7):634-45.

30. Shim SR, Shin IS, Bae JM. Intervention meta-analysis using STATA software. J Health Info Stat 2016;41(1):123-34. (korean)

31. Balding DJ. A tutorial on statistical methods for population association studies. Nat Rev Genet 2006;7(10):781-91.

32. Moonesinghe R, Khoury MJ, Liu T, Ioannidis JP. Required sample size and nonreplicability thresholds for heterogeneous genetic associations. Proc Natl Acad Sci USA. 2008;105(2):617-22.

33. Kavvoura FK, Ioannidis JP. Methods for meta-analysis in genetic association studies: a review of their potential and pitfalls. Hum Genet 2008;123(1):1-14.

34. Munafò MR, Clark TG, Flint J. Assessing publication bias in genetic association studies: evidence from a recent meta-analysis. Psychiatry Res 2004;129(1):39-44.

35. Shim SR, Shin IS, Yoon BH, Bae JM. Dose-response meta-analysis using STATA software. J Health Info Stat 2016;41(3):351-8. (korean)

36. Begum F, Ghosh D, Tseng GC, Feingold E. Comprehensive literature review and statistical considerations for GWAS meta-analysis. Nucleic Acids Res 2012;40(9):3777-84.

37. Wise LH, Lanchbury JS, Lewis CM. Meta-analysis of genome searches. Ann Hum Genet 1999;63(Pt 3):263-72.

38. Gonzalez de Castro D, Clarke PA, Al-Lazikani B, Workman P. Personalized cancer medicine: molecular diagnostics, predictive biomarkers, and drug resistance. Clin Pharmacol Ther

2013;93(3):252-9.

39. Dammann O, Gray P, Gressens P, Wolkenhauer O, Leviton A. Systems epidemiology: what's in a name? Online J Public Health Inform 2014;6(3):e198.

한글요약

오늘날 활발히 수행되고 있는 유전체역학연구는 재현성의 문제, 대상자 크기의 한계 등으로 유전체 메타분석 연구가 요구되고 있다. 유전체 메타분석의 전반적인 수행 단계는 약물, 수술 등의 중개연구의 메타분석처럼 5 단계를 밟아 수행된다. 그러나, 유전체 메타분석은 3번째 과정에서 Hardy–Weinberg equilibrium (HWE) 검정과 4번째 과정에서 5가지 가능한 유전형 모델에 따라 메타분석이 이루어진다는 점에서 중개연구의 메타분석과 차이가 있다.
